# Biomarkers for the Discrimination of Acute Kawasaki Disease From Infections in Childhood

**DOI:** 10.3389/fped.2020.00355

**Published:** 2020-07-22

**Authors:** Judith Zandstra, Annemarie van de Geer, Michael W. T. Tanck, Diana van Stijn-Bringas Dimitriades, Cathelijn E. M. Aarts, Sanne M. Dietz, Robin van Bruggen, Nina A. Schweintzger, Werner Zenz, Marieke Emonts, Dace Zavadska, Marko Pokorn, Effua Usuf, Henriette A. Moll, Luregn J. Schlapbach, Enitan D. Carrol, Stephane Paulus, Maria Tsolia, Colin Fink, Shunmay Yeung, Chisato Shimizu, Adriana Tremoulet, Rachel Galassini, Victoria J. Wright, Federico Martinón-Torres, Jethro Herberg, Jane Burns, Michael Levin, Taco W. Kuijpers, EUCLIDS Consortium, PERFORM Consortium and UK Kawasaki Disease Genetics Study Network

**Affiliations:** ^1^Sanquin Research and Landsteiner Laboratory, Department of Immunopathology, Amsterdam University Medical Centers, University of Amsterdam, Amsterdam, Netherlands; ^2^Sanquin Research and Landsteiner Laboratory, Department of Blood Cell Research, Amsterdam University Medical Centers, University of Amsterdam, Amsterdam, Netherlands; ^3^Department of Clinical Epidemiology, Biostatistics and Bioinformatics, Amsterdam UMC, University of Amsterdam, Amsterdam, Netherlands; ^4^Department of Pediatric Immunology, Rheumatology and Infectious Diseases, Emma Children's Hospital, Amsterdam UMC, University of Amsterdam, Amsterdam, Netherlands; ^5^Department of General Pediatrics and Adolescent Medicine, Medical University of Graz, Graz, Austria; ^6^Pediatric Infectious Diseases and Immunology Department, Great North Children's Hospital, Newcastle upon Tyne Hospitals NHS Foundation Trust, Newcastle upon Tyne, United Kingdom; ^7^Department of Pediatrics, Riga Stradins University, Riga, Latvia; ^8^Department of Infectious Diseases, University Medical Centre Ljubljana, Ljubljana, Slovenia; ^9^Medical Research Council Unit the Gambia (MRCG) at LSHTM, Serrekunda, Gambia; ^10^Department of General Pediatrics, Erasmus MC—Sophia Children's Hospital, Rotterdam, Netherlands; ^11^Pediatric Intensive Care Unit, Lady Cilento Children's Hospital, Pediatric Critical Care Research Group, Brisbane, QLD, Australia; ^12^Department of Clinical Infection, Microbiology and Immunology, University of Liverpool Institute of Infection and Global Health, Liverpool, United Kingdom; ^13^Second Department of Pediatrics, P. & A. Kyriakou Children's Hospital, National and Kapodistrian University of Athens, Athens, Greece; ^14^Micropathology Ltd., University of Warwick, Warwick, United Kingdom; ^15^Department of Clinical Research, Faculty of Infectious and Tropical Disease, London School of Hygiene and Tropical Medicine, London, United Kingdom; ^16^Section of Paediatric Infectious Diseases, Department of Infectious Disease, Imperial College London, London, United Kingdom; ^17^Kawasaki Disease Research Center, Rady's Children's Hospital—San Diego, University of California, San Diego, San Diego, CA, United States; ^18^Translational Pediatrics and Infectious Diseases, Hospital Clínico Universitario de Santiago, University of Santiago, Santiago de Compostela, Spain

**Keywords:** kawasaki disease, infectious disease, vasculitis, coronary aneurysm, biomarker, bacterial infection, viral infection

## Abstract

**Background:** Kawasaki disease (KD) is a vasculitis of early childhood mimicking several infectious diseases. Differentiation between KD and infectious diseases is essential as KD's most important complication—the development of coronary artery aneurysms (CAA)—can be largely avoided by timely treatment with intravenous immunoglobulins (IVIG). Currently, KD diagnosis is only based on clinical criteria. The aim of this study was to evaluate whether routine C-reactive protein (CRP) and additional inflammatory parameters myeloid-related protein 8/14 (MRP8/14 or S100A8/9) and human neutrophil-derived elastase (HNE) could distinguish KD from infectious diseases.

**Methods and Results:** The cross-sectional study included KD patients and children with proven infections as well as febrile controls. Patients were recruited between July 2006 and December 2018 in Europe and USA. MRP8/14, CRP, and HNE were assessed for their discriminatory ability by multiple logistic regression analysis with backward selection and receiver operator characteristic (ROC) curves. In the discovery cohort, the combination of MRP8/14+CRP discriminated KD patients (*n* = 48) from patients with infection (*n* = 105), with area under the ROC curve (AUC) of 0.88. The HNE values did not improve discrimination. The first validation cohort confirmed the predictive value of MRP8/14+CRP to discriminate acute KD patients (*n* = 26) from those with infections (*n* = 150), with an AUC of 0.78. The second validation cohort of acute KD patients (*n* = 25) and febrile controls (*n* = 50) showed an AUC of 0.72, which improved to 0.84 when HNE was included.

**Conclusion:** When used in combination, the plasma markers MRP8/14, CRP, and HNE may assist in the discrimination of KD from both proven and suspected infection.

## Introduction

Kawasaki disease (KD) is a systemic vasculitis of early childhood occurring mainly in children under the age of 5 years. The origin of the disease is still unknown. The current paradigm is that KD is caused by an immunologic reaction elicited by an (infectious) trigger in genetically predisposed children. KD's most important complication is the development of coronary artery aneurysms (CAA), affecting 25% of the untreated patients (1). This makes KD the most common cause of acquired heart disease in developed countries in children. The risk of CAA development is decreased 5-fold when the patient is treated with high-dose intravenous immunoglobulin (IVIG) and oral aspirin within 10 days of disease onset (1).

No specific diagnostic laboratory test for KD is available to date and KD is diagnosed based on the presence of clinical criteria, including prolonged fever, rash, lymphadenopathy, conjunctival injection, and abnormalities of mucosae and extremities. However, KD can be easily misdiagnosed due to the symptomatology resembling several infections (1–3) and the fact that bacterial and viral pathogens are regularly found in KD patients (2). Additionally, atypical or incomplete KD may also hinder the diagnosis. Therefore, KD diagnosis and initiation of treatment are commonly delayed, with a resulting increased risk of CAA development.

A rapid blood test to distinguish KD from infection, which enables the diagnosis and early treatment initiation, would reduce the burden of KD-related heart disease. Up to now, studies on surrogate plasma markers for acute KD have not included adequate febrile infection controls (4–6). While previous studies have shown promise that KD patients can be discriminated from febrile controls using gene expression (5), findings based on proteins may be more easily translatable into tests with immediate clinical utility.

We selected plasma biomarkers shown to be increased in KD patients and combined these markers to investigate whether a multi-protein test would discriminate KD from infection. We focused on C-reactive protein (CRP) and two neutrophil-derived proteins, myeloid-related protein 8/14 (MRP8/14) and human neutrophil elastase (HNE). MRP8/14 (S100A8/9 or calprotectin), a cytoplasmic protein found in neutrophils, and to a much lesser extent in monocytes, belongs to a family of calcium-binding proteins (7). MRP8/14 is released into the extracellular space upon cell activation, operating as a damage-associated molecular pattern (DAMP). MRP8/14 is recognized as a biomarker for several inflammatory diseases, including inflammatory bowel disease, and rheumatoid arthritis (8–10). In acute KD patients, elevated MRP8/14 levels have been previously reported, but in studies with no febrile controls (4, 11, 12). HNE, a well-known marker of neutrophil activation, is a neutrophil-specific serine protease located in the neutrophil azurophilic granules. HNE has been shown to be increased in the acute phase of KD compared to afebrile healthy controls (13).

CRP, an acute inflammatory protein that is synthesized in hepatocytes, binds to polysaccharides on microorganisms, triggering the classical complement pathway and initiating an innate immune response. CRP is commonly used as a biomarker as it is elevated during both acute infection and inflammation (14, 15).

The aim of this study was to evaluate whether MRP8/14, CRP, or HNE may act as possible biomarker(s) to diagnose acute (including incomplete) KD in febrile children. We analyzed the MRP8/14 levels before and after treatment in paired samples available from KD patients and investigated whether MRP8/14, CRP, and/or HNE could be used as a marker for the prediction of responsiveness to IVIG treatment and/or of CAA development during acute KD.

## Methods

We have used three cohorts of KD patients: a discovery cohort and two independent validation cohorts. For comparison, we used prospective samples collected in independent studies from febrile children diagnosed with acute bacterial and viral infections as well as those suspected of infection.

### KD Patients

Children (<18 years old) with acute KD based on the criteria of the American Heart Association (16) were recruited at the University Medical Center in Amsterdam [Amsterdam UMC, location Academic Medical Center (AMC)] and participating hospitals in the Netherlands as approved by the Medical Ethical Board of the AMC (no. NL41023.018.12) (for the discovery cohort). Patients were recruited between July 2006 and December 2018. For our first validation cohort, KD samples were included from the ongoing UK Kawasaki study “*Genetic determinants of Kawasaki Disease for susceptibility and outcome*” (13/LO/0026). This study recruits acutely unwell children with KD during hospital admission in participating hospitals around the UK. Our second validation cohort included KD patients who met the American Heart Association criteria for complete or incomplete KD cared for at Rady Children's Hospital, San Diego, CA.

The medical records of KD patients were reviewed and the clinical details recorded. CAA were defined by worst ever *z* scores: CA dimensions as standard deviation units normalized for body surface area (17). CAA was defined as a coronary *z* score ≥2.5.

EDTA plasma samples of the KD discovery cohort were collected before and, if available, after IVIG treatment. All samples taken within 14 days from disease onset (fever) were selected for the analysis and only those within 2 days after the start of IVIG were included as “acute,” except for the analysis of the paired samples of “acute disease” and convalescent after IVIG, from which the samples were taken during convalescence up to 1 year after the onset of disease. In the validation cohorts, samples from the “acute disease” were all samples before IVIG was administered.

### Febrile Patients With Definite Infections

For the discovery and the first validation cohorts, we compared KD patients to children presenting with acute febrile illness caused by a bacterial or viral illness. All children with a bacterial infection had a microbiologically confirmed pathogen detected in a normally sterile site and a consistent corresponding syndrome, including sepsis, meningitis, osteomyelitis, or pneumonia. All children with a viral illness had a detected viral pathogen, confirmed with culture, molecular, and/or immunofluorescent testing, and a consistent corresponding syndrome, without hallmarks of bacterial illness. In the viral infection group, a CRP of <60 was used to define a set of patients with a confident viral infection, as described in (18).

#### Discovery cohort

EDTA plasma samples from children (<18 years old) were collected at the first available time after presentation (within 48 h), in European hospitals participating in the EUCLIDS Consortium (19) (EU-Childhood Life-Threatening Infectious Disease Study; www.euclids.eu) and in the GENDRES (GENetic, Vitamin D and RESpiratory Infections Research Network; www.gendres.org) (20). Patients were included between December 2009 and May 2014. Clinical details were recorded as part of the study, including gender, age at disease onset, hospitalization, and details about the type of disease and the invasive pathogen involved when cultured from sterile sites.

#### First Validation Cohort

Patients were included after local approval in the international study on febrile children (PERFORM; https://www.perform2020.org/). Overall, patients were included between July 2012 and December 2018. EDTA plasma samples were collected at presentation to hospital in the Emergency Department or ward. Clinical details were recorded as described above.

### Febrile Patients With Suspected Infections

For the second validation cohort, we compared KD patients to children presenting with acute febrile illness with suspected infection.

#### Second Validation Cohort

Febrile control patients were recruited from the Emergency Department at Rady Children's Hospital, San Diego, CA. The study protocol was reviewed and accepted by the UCSD institutional review board. Parental consent was obtained, where appropriate. The inclusion criteria for the febrile controls were fever for at least 3 days, no use of steroids, and at least one clinical sign of KD. Review of the medical records at least 1 month after the onset of fever retrospectively confirmed that these patients all had a self-limited illness that required no anti-infective treatment and were deemed likely due to a non-specific viral illness. The majority of these patients had been initially referred to the hospital to rule out KD.

### Healthy Controls

After informed consent, EDTA-anticoagulated blood samples from healthy volunteer donors were obtained *via* an internal system at Sanquin (the Dutch national blood bank) after consulting the Medical Ethical Committee from the Academic Medical Center Amsterdam. All procedures were conducted in accordance with the 1975 Declaration of Helsinki as revised in 2013, and local ethics approval were obtained within the PERFORM Study mentioned above. Median age was 37.8 (interquartile range, IQR = 37.8–58.7) years and 39.5% were male. No clear age-defined or gender-related effects were seen in the baseline values (13).

### Analysis of Neutrophil Activation Markers

MRP8/14 was measured in a new enzyme-linked immunosorbent (ELISA) assay developed at Sanquin. All washes between the incubation steps were done with phosphate-buffered saline (PBS) 0.02% Tween-20 using Elx 405 (BioTek Instruments). Anti-human MRP8/14 monoclonal mouse IgG1 antibody, clone 27E10 (0.5 μg/ml in 0.1 M carbonate buffer, pH 9.6; BMA Biomedicals, Augst, Switzerland), was used to coat a Maxisorp Nunc-immunoplate (Thermo Scientific). After overnight static incubation at room temperature and wash, serum pooled from 30 healthy donors was added to give a calibration curve [diluted 2-fold from 8.5 ng/ml (*v*/*v*) in TTG (10 mM Tris, 150 mM NaCl, 10 mM CaCl_2_, 0.1% Tween-20, 0.2% gelatin, pH 7.4)]. As a positive control, the pooled sera was spiked with 10 μg/ml recombinant MRP8/14 (Hycult Biotech, Plymouth Meeting, PA, USA) and diluted to 0.1% (*v*/*v*). The samples, positive control, and calibration curve were incubated for 1 h. After washing, 500 ng/ml biotinylated mouse monoclonal IgG1 anti-MRP8/14 antibody, clone S36.48 (BMA Biomedicals), was added. After incubation for 1 h, further washing, and the addition of streptavidin conjugated to poly-horseradish peroxidase [poly-HRP, 0.1% (*v*/*v*)] for 30 min followed by 100 μg/ml 3,3′,5,5′-tetramethylbenzidine (TMB; Merck Chemicals) in 0.11 M sodium acetate, pH 5.5, with 0.003% (*v*/*v*) H_2_O_2_, and 2 M H_2_SO_4_, absorbance was measured at 450/540 nm. The results are expressed in nanograms per milliliter by reference of the calibration curve.

Plasma HNE is not present in blood alone, but in complex with α1-antitrypsin (α1AT). Therefore, plasma HNE was measured as HNE–α1AT complexes. CRP and HNE–α1AT complexes were determined by ELISAs, as described previously (21, 22).

### Statistical Analysis

Data were assessed to determine normal distribution. Differences in the MRP8/14, CRP, and HNE values between the patient cohorts were plotted as the median + IQR. Log_10_-transformed data were used with respect to linearity for further statistical analyses on the predictive values of the markers.

A logistic regression analysis with backward selection was used to examine the predictive value (odds ratio) of the candidate markers for acute KD vs. infections and for KD vs. bacterial and KD vs. viral infections.

Receiver operating characteristic (ROC) curves were plotted from the predictive markers derived from the logistic regression analysis. The area under the ROC curve (AUC) from both the individual candidate markers as well as a combination of candidate markers (the predicted probability) was used to check for discriminatory value. The best discriminatory cutoff value was calculated with Youden's J Statistic. The cutoff from the discovery cohort was applied to the validation cohorts and the corresponding sensitivity and specificity with 95% confidence intervals (CI) were calculated. Using predicted probabilities based on the discovery model, ROC curves in the validation cohorts were plotted and AUCs were compared to the AUC's from the discovery cohort.

For the analysis of the MRP8/14 levels pre- and post-IVIG treatment, a Wilcoxon signed-rank test was performed in paired samples. The predictive capacities of the markers for treatment response and CAA development in acute KD patients were calculated by a logistic regression with backward selection.

A *p* < 0.05 was considered to be statistically significant. SPSS version 24.1 (IBM, Armonk, NY) and R version 3.4.4 (The R Foundation for Statistical Computing, Vienna, Austria) were used for all statistical analyses.

## Results

Our discovery cohort consisted of 48 patients with acute KD (27 pre-IVIG treatment and 21 shortly after IVIG treatment had been administered, within 3 days). Of these acute patients, 10 were diagnosed with incomplete KD. In total, 105 patients were included with infections (40 patients with viral infections and 65 patients with bacterial infections). Our first validation cohort consisted of 26 patients with acute KD (pre-IVIG treatment) and 150 patients with infections (75 bacterial and 75 viral) diagnosed at different centers in Europe. The second validation cohort consisted of 50 KD patients (with paired measurements pre- and post-IVIG treatments, i.e., acute vs. convalescent disease) and 50 febrile control patients. The demographic and clinical characteristics of all patients are shown in Table 1. Supplemental Table 1 provides a more detailed overview of the patients with definite (i.e., proven) infections in our discovery and first validation cohorts.

**Table 1:** Demographic and clinical characteristics of the patients in the acute Kawasaki disease, bacterial, and viral infections cohort and in the discovery and validation cohorts.

	**Discovery cohort**			**First validation cohort**			**Second validation cohort**		
	**Kawasaki disease, *n* = 48 (27 pre-IVIG, 21 post-IVIG)**	**Bacterial infections, *n* = 65**	**Viral infections, *n* = 40**	**Kawasaki disease, *n* = 26**	**Bacterial infections, *n* = 75**	**Viral infections, *n* = 75**	**Kawasaki disease, pre-IVIG, *n* = 50**	**Kawasaki disease, post-IVIG, *n* = 50**	**Febrile control, *n* = 50**
% Male (*n*)	64.4% (31)	47.7% (31)	45% (18)	69.2% (18)	54.7% (41)	57.3% (43)	46% (23)	46% (23)	48% (24)
Age at sample date (years, median +)	2.1 (1.1–4.5)	9 (3–14.3)	1.4 (0.2–3.6)	2.8 (1–3.8)	4.58 (0.8–7.5)	3 (0.8–6.9)	2.7 (1.6–5.5)	2.7 (1.6–5.5)	3.4 (1.2–5.2)
Days of fever before hospital admission (days, median +)	8 (6–11)	1 (0–2)	1 (0–2)	6 (5–8)	1 (1–5)	2 (1–5)	5 (4–7)	19 (17–21)	5 (4–7)
Days until IVIG treatment (days)	<10 days	Not applicable	Not applicable	<10 days	Not applicable	Not applicable	<10 days	Notapplicable	Not applicable
% CAA (*n*)	CAA: 25% (12) Giant: 8.3% (4)	Not applicable	Not applicable	CAA: 15.4% (4) Giant: 0%	Not applicable	Not applicable	CAA: 24% (12) Giant: 0%	CAA: 24% (12) Giant: 0%	Not applicable
% Unresponsive to first IVIG course (*n*)	25% (12)	Not applicable	Not applicable	23% (6)	Not applicable	Not applicable	4% (4)	Not applicable	Not applicable
% Ethnicity	Caucasian: 68.6%; Asian: 4.2%; Other: 4.2%; Unknown: 23%	Caucasian: 80%; Asian: 1.5%; Other: 4.5%; Unknown: 14%	Caucasian: 62.5%; Asian: 12.5%; Hispanic: 12.5%; Other: 12.5%	Caucasian: 23%; Asian: 11.5%; African: 34.5%; Other: 15.5%; Unknown: 15.5%	Caucasian: 53.3%; Asian: 1.3%; Hispanic: 21.3%; Mixed: 1.3%; Other: 10.6%	Caucasian: 56%; Asian: 10.6%; Hispanic: 21.3%; Mixed: 1.3%; Other: 10.6%	Caucasian: 12%; Asian: 18%; Hispanic: 42%; Mixed: 28%	Caucasian: 12%; Asian: 18; Hispanic: 42%; Mixed: 28%	Caucasian: 20%; Asian: 8%; Hispanic: 42%; Mixed: 22%; Unknown: 8%

*Giant aneurysm is defined by a z score >2.5*.

*CAA, coronary artery aneurysm*.

### MRP8/14 and CRP Discriminate Between Acute KD and an Infection

**Figure 1:**
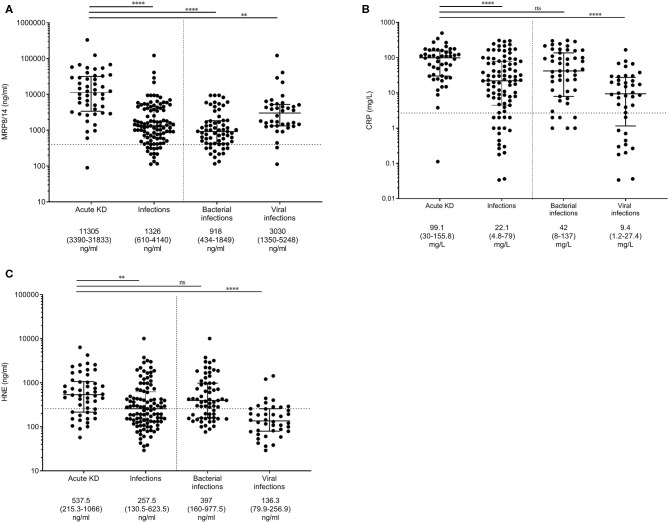
Myeloid-related protein 8/14 (MRP8/14) **(A)**, C-reactive protein (CRP) **(B)**, and neutrophil-derived elastase (HNE) **(C)** levels in the discovery cohort. Patients with acute Kawasaki disease are compared to patients with infections and, more specifically, patients with either bacterial or viral infections. Each *dot* is a unique patient. *Dotted line* represents the 75% concentration in the healthy controls: 400 ng/ml MRP8/14, 2.7 mg/l CRP, and 37.3 ng/ml HNE. Median levels + interquartile ranges are listed *below the figure*. Differences were calculated with the Kruskal–Wallis test, followed by Dunn's multiple comparisons test. **p < 0.005, ****p < 0.001; *ns*, not significant.

First, we aimed to determine the differences in MRP8/14, CRP, and HNE in the discovery cohort (Figures 1A–C). MRP8/14, CRP, and HNE were significantly different between the KD and infectious patients. The results from our two validation cohorts are shown in Supplemental Figure 1. When assessing KD vs. bacterial infections only, the MRP8/14 levels were significantly elevated in KD. In the bacterial patients, a high- and low-MRP8/14 group could be distinguished. The patient group with the high MRP8/14 levels did not have a pathogen or diagnosis in common. No correlation was found between the MRP8/14 or HNE and absolute neutrophil counts (data not shown).

Multiple logistic regression analysis with backward selection on the discovery cohort showed that elevated levels of MRP8/14 (log_10_ OR = 7.7, 95% CI = 3.2–18.5) and CRP (log_10_ OR = 5.1, 95% CI = 1.7–14.7) were predictive for acute KD over an infection (*p* < 0.001). HNE was removed from the analysis during backward selection.

**Figure 2:**
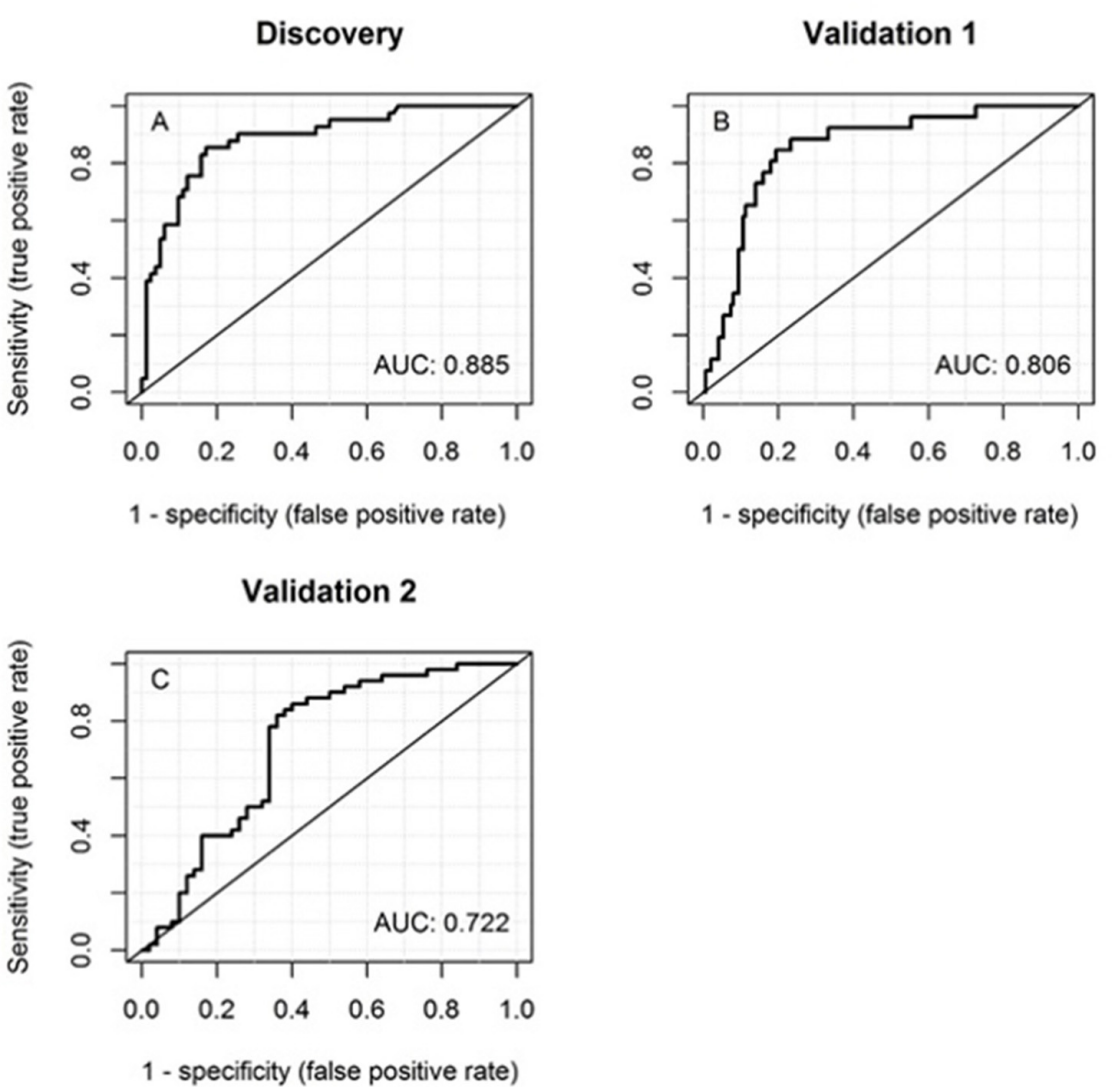
Receiver operator characteristic (ROC) curves of the discovery cohort **(A)**, first validation cohort **(B)**, and the second validation cohort **(C)** of MRP8/14 and CRP together for discrimination between acute Kawasaki disease and infections. The corresponding areas under the ROC curve (AUCs) are listed *within the panels*.

ROC curves were plotted for the predictive ability of MRP8/14 and CRP to discriminate between acute KD and an infection. The AUC for MRP8/14 was 0.86 (95% CI = 0.78–0.93, *p* < 0.001), with a sensitivity of 70% and a specificity of 91% and cutoff of 6,540 ng/ml. For CRP, the AUC was 0.76 (95% CI = 0.68–0.85, *p* < 0.001), with a sensitivity of 78% and a specificity of 67% and cutoff of 43.9 mg/l. The ROC curve of the predicted probability of KD when MRP8/14 and CRP were used together was 0.88 (95% CI = 0.82–0.95, *p* < 0.001), with a sensitivity of 85% and a specificity of 83% (Figure 2A). The optimal cutoff from the discovery cohort (predicted KD probability of 0.37 or higher) was applied to the first validation cohort and the corresponding sensitivity and specificity were determined, showing a sensitivity of 73% and a specificity of 77%. The ROC curve from the predicted probability of KD when both MRP8/14 and CRP were used in the first validation cohort showed an AUC of 0.81 (95% CI = 0.73–0.88) (Figure 2B).

The same optimal cutoff was also applied to our second validation cohort, with patients from the USA. Here, we found a sensitivity of 74% and a specificity of 62%. The ROC curve with both MRP8/14 and CRP showed an AUC of 0.72 (95% CI = 0.62–0.82) (Figure 2C).

### Meta-Analysis of All Cohorts

In this study, we wanted to test the values of CRP, MRP8/14, and HNE in predicting KD over an infection. A summary of all the AUCs and the sensitivity and specificity values for MRP8/14+CRP in the different cohorts is shown in Supplemental Table 2. Three independent cohorts showed a strong predicted probability of KD over an infection when combining MRP8/14 and CRP. HNE was eliminated in the analysis after the logistic regression with backward selection in our discovery cohort. By analyzing all three cohorts together in a meta-analysis, we created a large cohort with a range of ethnicity. This meta-cohort was used to test our model and see whether revision or extension of the existing prediction model was necessary.

**Figure 3:**
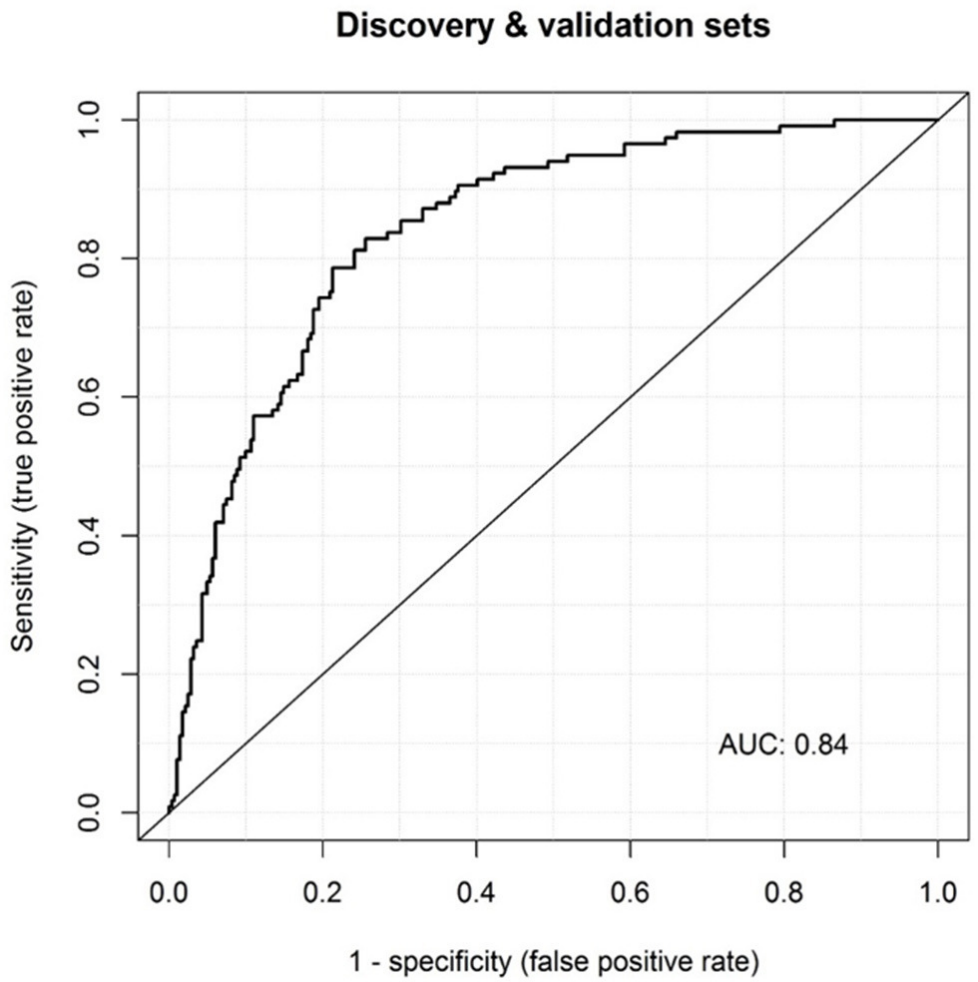
Receiver operator characteristic (ROC) curves of MRP8/14, CRP, and HNE combined in the meta-analysis. The corresponding area under the ROC curve (AUC) is listed *within the panel*.

Our large cohort consisted of 124 acute KD patients and 305 febrile patients (proven infections combined with the “undefined” febrile controls). We re-estimated the effects of our current model and investigated a possible extension with HNE in this large cohort. Multiple logistic regression analysis showed that elevated levels of MRP8/14 (log_10_ OR = 5.5, 95% CI = 3.1–9.6), CRP (log_10_ OR = 2.4, 95% CI = 1.3–4.1), and HNE (log_10_ OR = 2.3, 95% CI = 1.3–4.0) were predictive of acute KD over an infection (*p* < 0.001). Although HNE was not included in the initial model, in our large cohort, we saw a significant effect of HNE in predicting KD over an infection. The discriminatory power of the model with MRP8/14, CRP, and HNE showed an AUC of 0.84 (95% CI = 0.80–0.88), with a sensitivity of 74% and a specificity of 83% (Figure 3).

### MRP8/14 as a Marker for Treatment Response and CAA Development

Prediction of IVIG resistance and risk of developing CAA by biomarkers would greatly aid clinical decision making. In the discovery cohort and the second validation cohort, we compared the levels of MRP8/14 in paired samples and in those with and without CAA.

**Figure 4:**
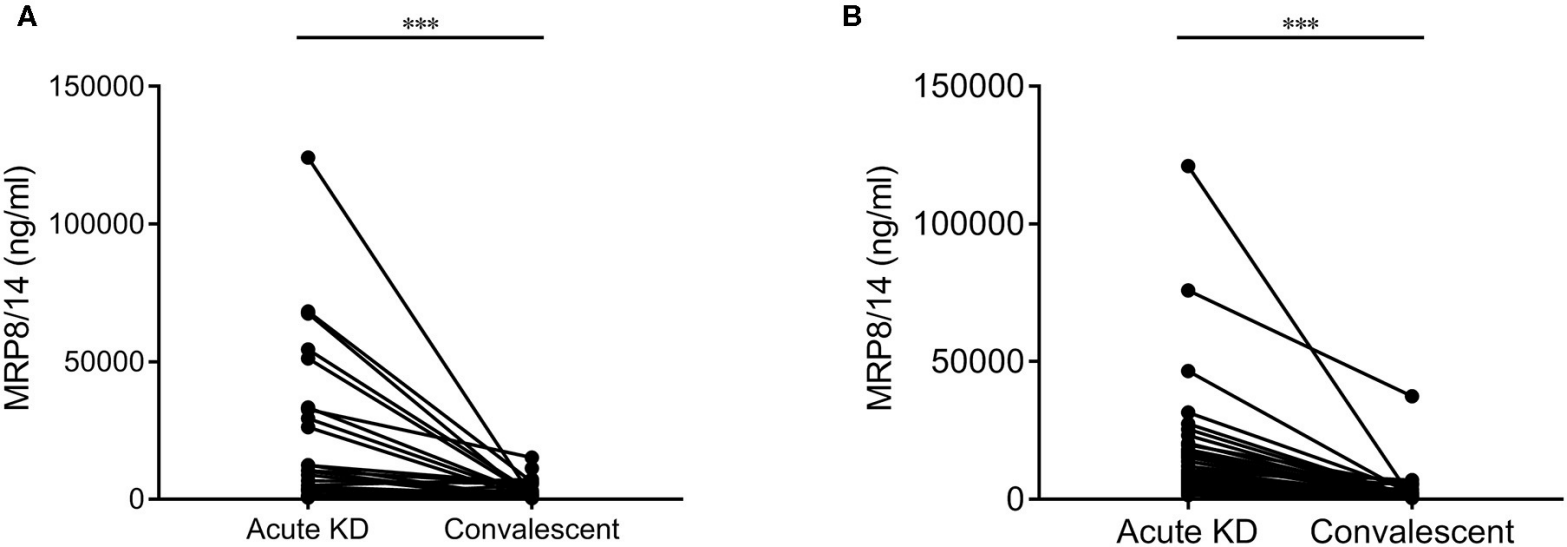
Differences in the MRP8/14 levels in acute Kawasaki disease (KD) and convalescent in 25 paired samples from the discovery cohort **(A)** and 50 paired samples from the second validation cohort **(B)**. In the discovery cohort, one sample above 30,000 ng/ml MRP8/14 was excluded in this graph. Analyzed by a Wilcoxon matched-pairs test. ***p < 0.0001.

A total of 25 children had paired measurements pre-IVIG treatment and during convalescence [median of 49 days (IQR = 27–155 days) after disease onset]. The samples collected prior to IVIG administration showed significantly higher levels of MRP8/14 (median = 10,400 ng/ml, IQR = 3,500–42,500 ng/ml) compared to the samples collected during post-IVIG convalescence (median = 1,600 ng/ml, IQR = 300–5,600 ng/ml, *p* < 0.0001) (Figure 4A).

In our second validation cohort, a total of 50 patients had paired measurements before IVIG treatment and during convalescence [median of 20 days (IQR = 17–22 days) after disease onset]. Again, the samples pre-IVIG treatment showed significantly higher levels of MRP8/14 (median = 8,398 ng/ml, IQR = 4,506–16,025 ng/ml) compared to the post-IVIG convalescence state (median = 836 ng/ml, IQR = 450.8–1,924 ng/ml, *p* < 0.0001) (Figure 4B). The same significant decrease was seen in CRP and HNE in both the discovery and validation cohorts (data not shown). A logistic regression analysis showed that neither MRP8/14 nor one of the other markers was predictive of clinical IVIG response.

As CAA are the most prevalent complications of KD, we combined all our KD samples and assessed whether the MRP8/14 values were elevated in patients with CAA, but no significant differences were seen (Supplemental Figure 2). Also, a logistic regression analysis showed that none of the markers was predictive of CAA development (data not shown).

## Discussion

To date, a rapid protein test to distinguish KD from infection is not available. Different studies have been conducted in the search for a biomarker, but have failed to find a single and reliable biomarker. This includes studies on a broad panel of routinely used parameters (23), antibody profiling using of *Escherichia coli* proteome microarrays, (24), or the identification of candidate biomarkers using multiplex cytokine and protein arrays (25). In this study, we have investigated inflammation-related plasma-derived biomarkers to identify pediatric patients with acute KD among their febrile peers with a proven or suspected infection. Our most important finding was that a combined measurement of MRP8/14 and CRP, and possible HNE, was indicative of KD, discriminating KD from probable infections in febrile children. When applying the optimal cutoffs to the validation cohorts, we observed an impaired specificity of the markers, with an overestimation of KD. This might be a result of the differences in pathogens (Supplemental Table 1). The different case/control ratios in the three cohorts could possibly influence the estimated sensitivities, specificities, and the derived AUCs, but these effects are not systemic (26). In the discovery cohort, we included 21 KD patients in the acute group, up to 3 days after IVIG treatment. We split the KD group into pre-IVIG treatment and shortly after IVIG treatment and compared the levels of MRP8/14, CRP, and HNE in these two subgroups. There was no difference seen (data not shown), showing that these biomarkers are stable in the acute phase of KD up to 3 days after IVIG treatment. What we have noticed before is that HNE remains detectable in KD when measured at regular time points up to 3 months following the acute stage (13) and that CRP remains elevated in KD patients with CAA more than 5 years after disease onset (27). This indicates that low-grade inflammation may indeed be present subclinically much longer than previously thought.

It is highly encouraging that candidate plasma markers for acute KD in febrile children have been found and validated against common bacterial and viral infections. Together with the clinical manifestations at presentation of these patients, the combined use of these markers will enable physicians to be more confident in their KD diagnosis. Although MRP8/14 is significantly lower after treatment with IVIG in the paired samples, its pretreatment levels could not predict clinical IVIG response in our discovery cohort and in the second validation cohort. We also did not find a correlation of the MRP8/14 (nor CRP and HNE) levels with CAA.

The most important consequence of a quick and correct KD diagnosis is the ability to promptly initiate IVIG treatment and, thereby, reduce the risk of CAA development. CRP is used, but is non-discriminatory. HNE has been used before in a previous study of our group by (13). A few studies have reported on the MRP8/14 levels in children with KD. Hirono et al. found increased MRP8/14 concentrations before IVIG as compared to post-IVIG in 45 IVIG-responding KD patients, whereas the levels did not decrease after IVIG in 16 IVIG non-responders (12). Four weeks after IVIG treatment, these patients still showed higher MRP8/14 concentrations compared to IVIG responders. Abe et al. found increased MRP8/14 levels in 32 KD patients before IVIG treatment compared to after IVIG treatment and compared to controls with fever of unknown origin (4), which may correlate with the epigenetic regulation of S100A genes (28). Viemann et al. found that the MRP8/14 concentrations dropped significantly within 24 h after IVIG treatment in 21 KD patients (11). After 1 month, the concentrations reached the values of their healthy controls. These authors found that the endothelium of the coronary arteries from the myocardial sections of three deceased KD patients were almost completely coated when stained with antibodies against MRP8 or MRP14 (S100A8 or S100A9). Recent investigation using a proteomic approach documented elevated levels of MRP8/14 decades after acute KD in patients with giant aneurysms (29). The variations in the correlations with CAA development may depend on technical differences in measuring CAA dimensions, but more likely will be determined by statistical power (or lack thereof) because of the small sample size of these studies, the non-prospective study design, and maybe also because of genetic differences in the cohorts tested. A conclusion as to whether the biomarker(s) really have predictive values will demand prospectively designed large studies to settle the diverse findings to date.

There are indications that MRP8/14 is a regulator of vascular inflammation (30) and is found to be elevated during different types of vasculitis (31, 32) and during vascular injury (33, 34). Furthermore, MRP8/14 has been shown to impair endothelial integrity (35), and high amounts of MRP8/14 in the coronary endothelium of KD patients are observed (11). Apart from acting as a DAMP, the special role of MRP8/14 during vascular inflammation could be a reason for its remarkable elevation during the acute vasculitis of KD. MRP8/14 is released from phagocytes after contact with the inflamed endothelium (36) and might also be released from the endothelium itself.

In our study, we have found that MRP8/14 and CRP, and possibly HNE, could be potentially used to help physicians diagnose KD in febrile children, enabling prompt treatment and lowering the risk of CAA formation. We were able to substantiate our findings in the validation cohorts, collected according to international diagnostic criteria. Additional prospective studies should now follow to more routinely perform our model of markers in pediatric patients with prolonged fever to further confirm our findings. The value of the combined tests may also prove to be helpful in the case of incomplete KD to decide whether IVIG treatment seems justified instead of further clinical observation waiting for microbiological results or the effect of antibiotics.

## Data Availability Statement

The datasets generated for this study are available on request to the corresponding author.

## Ethics Statement

The studies involving human participants were reviewed and approved by Medical Ethical Board of the AMC (# NL41023.018.12). Written informed consent to participate in this study was provided by the participants' legal guardian/next of kin.

## Author Contributions

JZ, AG, DS-B, CA, and SD collected data, performed data analyses, and wrote the initial manuscript. MWTT performed data analyses and reviewed and revised the final manuscript. NS, CS, AT, RG, and VW enrolled the patients, collected patient data, and reviewed and revised the final manuscript. RB, WZ, ME, DZ, MP, EU, HM, LS, EC, SP, MT, CF, SY, FM-T, JH, JB, and ML designed the study and reviewed and revised the final manuscript. TK coordinated and supervised data collection, designed the study, and reviewed and revised the final manuscript. All authors approved the final manuscript as submitted and agreed to be accountable for all aspects of the work.

## Conflict of Interest

The authors declare that the research was conducted in the absence of any commercial or financial relationships that could be construed as a potential conflict of interest.
