# Supplementary material for: Biomarkers for the Discrimination of Acute Kawasaki Disease From Infections in Childhood
Source: Front Pediatr. 2020 Jul 22;8:355. doi: 10.3389/fped.2020.00355 (PMC7388698; doi:10.3389/fped.2020.00355)
Supplement: Supplementary file 1 [file Data_Sheet_1.docx]

|  | **Bacterial** | **Viral** |
| --- | --- | --- |
| **Discovery cohort** | *E. coli* (3) | Adenovirus (4) |
|  | *M. pneumoniae* (5) | Enterovirus (13) |
|  | *N. meningitidis* (19) | Influenza (6) |
|  | *S. aureus* (17) | Parechovirus (4) |
|  | *Str. pneumoniae* (14) | Rhinovirus (6) |
|  | *Str. pyogenes* (5) | Other (7) |
|  | Other (2) |  |
| **First validation cohort** | *Enterococcus faecalis* (4) | Adenovirus (10) |
|  | *E. coli* (22) | Enterovirus (4) |
|  | *H influenzae* (3) | Epstein-Barr Virus (7) |
|  | *N. meningitidis* (6) | Herpes Simplex (4) |
|  | *Ps. aeruginosa* (4) | Influenza A (11) |
|  | *S. aureus* (12) | Influenza B (5) |
|  | *Str. pneumoniae* (5) | Measles (4) |
|  | *Str. Pyogenes* (14) | Metapneumovirus (2) |
|  | Other (5) | Norovirus (2) |
|  |  | Parainfluenza (3) |
|  |  | Respiratory Syncytial Virus (7) |
|  |  | Rhinovirus (4) |
|  |  | Other (12) |
|  |  |  |

**Supplemental Table 1:** Detailed overview of the pathogens identified in patients from the discovery cohort and the first validation cohort.

| **MRP8/14 + CRP** | **AUC** | **Sensitivity (%)** | **Specificity (%)** |
| --- | --- | --- | --- |
| Discovery cohort | 0.88 | 85 | 83 |
| First validation cohort | 0.81 | 73 | 77 |
| Second validation cohort | 0.72 | 74 | 62 |
| All cohorts combined | 0.80 | 74 | 74 |

**Supplemental Table 2.** Summary AUC, sensitivity and specificity when combining MRP8/14 and CRP in different cohorts.
