# Supplementary material for: Biomarkers for the Discrimination of Acute Kawasaki Disease From Infections in Childhood
Source: Front Pediatr. 2020 Jul 22;8:355. doi: 10.3389/fped.2020.00355 (PMC7388698; doi:10.3389/fped.2020.00355)
Supplement: Supplementary file 2 [file Data_Sheet_2.pdf]

Imperial College partner (UK)
Members of the EUCLIDS Consortium at Imperial College London (UK)
Principal and co-investigators

Michael Levin (grant application, EUCLIDS Coordinator)
Dr. Lachlan Coin (bioinformatics)
Stuart Gormley (clinical coordination)
Shea Hamilton (proteomics)
Jethro Herberg (grant application, PI)
Bernardo Hourmat (project management)
Clive Hoggart (statistical genomics)
Myrsini Kaforou (bioinformatics)
Vanessa Sancho-Shimizu (genetics)
Victoria Wright (grant application, scientific coordination)

Consortium members at Imperial College
Amina Abdulla
Paul Agapow
Maeve Bartlett
Evangelos Bellos
Hariklia Eleftherohorinou
Rachel Galassini
David Inwald
Meg Mashbat
Stefanie Menikou
Sobia Mustafa
Simon Nadel
Rahmeen Rahman
Clare Thakker

EUCLIDS UK Clinical Network

Great North Children's Hospital, Newcastle; Dr M Emonts (PI), Rachel Agbeko, Kirsty Devine,
Poole Hospital NHS Foundation Trust, Poole: Dr S Bokhandi (PI), Sue Power, Heather Barham
Cambridge University Hospitals NHS Trust, Cambridge: Dr N Pathan (PI), Jenna Ridout, Deborah White, Sarah Thurston
University Hospital Southampton, Southampton: Prof S Faust (PI), Dr S Patel (co-investigator), Jenni McCorkell.
Nottingham University Hospital NHS Trust: Dr P Davies (PI), Lindsey Crate, Helen Navarra, Stephanie Carter
University Hospitals of Leicester NHS Trust, Leicester: Dr R Ramaiah (PI), Rekha Patel
Portsmouth Hospitals NHS Trust, London: Dr Catherine Tuffrey (PI), Andrew Gribbin, Sharon McCready
Great Ormond Street Hospital, London: Dr Mark Peters (PI), Katie Hardy, Fran Standing, Lauren O'Neill, Eugenia Abelake
King's College Hospital NHS Foundation Trust, London; Dr Akash Deep (PI), Eniola Nsirim
Oxford University Hospitals NHS Foundation Trust, Oxford Prof A Pollard (PI), Louise Willis, Zoe Young

Kettering General Hospital NHS Foundation Trust, Kettering: Dr C Royad (PI), Sonia White
Central Manchester NHS Trust, Manchester: Dr PM Fortune (PI), Phil Hudnott

SERGAS Partner (Spain)

Principal Investigators

Federico Martinón-Torres[1]
Antonio Salas[1,2]

GENVIP RESEARCH GROUP (in alphabetical order):

Fernando Álvez González[1], Ruth Barral-Arca[1,2], Xabier Bello[1,2], Miriam Cebey-López[1], María José Curras-Tuala[1,2], Natalia García[1], Luisa García Vicente[1], Alberto Gómez-Carballa[1,2], Jose Gómez Rial[1], Pilar Leboráns Iglesias[1], Federico Martinón-Torres[1], Nazareth Martinón-Torres[1], José María Martinón Sánchez[1], Belén Mosquera Pérez[1], Jacobo Pardo-Seco[1,2], Sara Pischedda[1,2], Irene Rivero Calle[1], Carmen Rodríguez-Tenreiro[1], Lorenzo Redondo-Collazo[1], , Antonio Salas[1,2], Sonia Serén Fernández[1].

[1] Translational Pediatrics and Infectious Diseases, Pediatrics Department, Hospital Clínico Universitario de Santiago, Santiago de Compostela, Spain, and GENVIP Research Group (www.genvip.org), Instituto de Investigación Sanitaria de Santiago, Galicia, Spain.
[2] Unidade de Xenética, Departamento de Anatomía Patolóxica e Ciencias Forenses, Instituto de Ciencias Forenses, Facultade de Medicina, Universidade de Santiago de Compostela, and GenPop Research Group, Instituto de Investigaciones Sanitarias (IDIS), Hospital Clínico Universitario de Santiago, Galicia, Spain
[3] Fundación Pública Galega de Medicina Xenómica, Servizo Galego de Saúde (SERGAS), Instituto de Investigaciones Sanitarias (IDIS), and Grupo de Medicina Xenómica, Centro de Investigación Biomédica en Red de Enfermedades Raras (CIBERER), Universidade de Santiago de Compostela (USC), Santiago de Compostela, Spain

PERFORM SPANISH CLINICAL NETWORK:

Pablo Rojo[1], J.Ruiz Contreras[1], Alba Palacios[1].
[1] Hospital Universitario 12 de Octubre; Madrid, Spain.

RSU Partner (Latvia)

Principal Investigator

Dace Zavadska[1,2]

Other RSU group authors (in alphabetical order):

Anda Balode[1,2], Arta Bārzdiņa[1,2], Dārta Deksne[1,2], Dace Gardovska[1,2], Dagne Grāvele[2], Ilze Grope[1,2], Anija Meiere[1,2], Ieva Nokalna[1,2], Jana Pavāre[1,2], Zanda Pučuka[1,2], Katrīna Selecka[1,2], Aleksandra Sidorova[1,2], Dace Svile[2], Urzula Nora Urbāne[1,2].

[1] Riga Stradins university, Riga, Latvia.
[2] Children clinical university hospital, Riga, Latvia.

Medical Research Council Unit The Gambia (MRCG) at LSHTM Partner

Principal Investigator

Effua Usuf

Additional Investigators

Kalifa Bojang

Akram Zaman
Fatou Secka
Suzanne Anderson
Anna RocaIsatou Sarr
Momodou Saidykhan
Saffiatou Darboe
Samba Ceesay
Umberto D’alessandro

Medical Research Council Unit The Gambia at LSHTM
P O Box 273,
Fajara, The Gambia

ERASMUS MC-Sophia Children’s Hospital

Principal Investigator
Henriëtte A. Moll[1]

Research group
Dorine M. Borensztajn[1], Nienke N. Hagedoorn[1], Leontien M.H. Roos[1], Clementien L. Vermont[2]

[1] Erasmus MC-Sophia Children’s Hospital, Department of General Paediatrics, Rotterdam, the Netherlands
[2] Erasmus MC-Sophia Children’s Hospital, Department of Paediatric Infectious Diseases & Immunology, Rotterdam, the Netherlands

Swiss Pediatric Sepsis Study

Principal Investigators: Luregn J Schlapbach, MD, FCICM [1,2,3,4], Philipp Agyeman, MD [1], Christoph Aebi, MD [1], Christoph Berger, MD [1,13]

Research group
Eric Giannoni, MD [5,6], Martin Stocker, MD [7], Klara M Posfay-Barbe, MD [8], Ulrich Heininger, MD [9], Sara Bernhard-Stirnemann, MD [10], Anita Niederer-Loher, MD [11], Christian Kahlert, MD [11], Paul Hasters, MD [12], Christa Relly, MD [13], Walter Baer, MD [14] for the Swiss Pediatric Sepsis Study

[1.] Faculty of Medicine, The University of Queensland, Brisbane, Australia
[2.] Department of Pediatrics, Inselspital, Bern University Hospital, University of Bern, Switzerland
[3.] Paediatric Critical Care Research Group, Mater Research Institute, University of Queensland, Brisbane, Australia
[4.] Paediatric Intensive Care Unit, Lady Cilento Children’s Hospital, Children’s Health Queensland, Brisbane, Australia
[5.] Service of Neonatology, Lausanne University Hospital, Lausanne, Switzerland
[6.] Infectious Diseases Service, Lausanne University Hospital, Lausanne, Switzerland
[7.] Department of Pediatrics, Children’s Hospital Lucerne, Lucerne, Switzerland

[8.] Pediatric Infectious Diseases Unit, Children's Hospital of Geneva, University Hospitals of Geneva, Geneva, Switzerland
[9.] Infectious Diseases and Vaccinology, University of Basel Children's Hospital, Basel, Switzerland
[10.] Children's Hospital Aarau, Aarau, Switzerland
[101] Division of Infectious Diseases and Hospital Epidemiology, Children's Hospital of Eastern Switzerland St. Gallen, St. Gallen, Switzerland
[12.] Department of Neonatology, University Hospital Zurich, Zurich, Switzerland
[13.] Division of Infectious Diseases and Hospital Epidemiology, and Children's Research Center, University Children's Hospital Zurich, Switzerland
[14.] Children's Hospital Chur, Chur, Switzerland

Liverpool Partner

Principal Investigators
Enitan Carrol[1,2]
Stéphane Paulus [1,2]

Research Group (in alphabetical order):
Rebecca Jennings[3], Joanne Johnston[3], Simon Leigh[1], Karen Newall[3],
[1] Department of Clinical Infection, Microbiology and Immunology, University of Liverpool Institute of Infection and Global Health , Liverpool, England
[2] Alder Hey Children's Hospital, Department of Infectious Diseases, Eaton Road, Liverpool, L12 2AP
[3] Alder Hey Children's Hospital, Clinical Research Business Unit, Eaton Road, Liverpool, L12 2AP

Newcastle partner

Principle Investigator
Marieke Emonts[1,2]

Co-Investigator
Rachel Agbeko[1,3]

[1] Institute of Cellular Medicine, Newcastle University, Newcastle upon Tyne, United Kingdom
[2] Paediatric Infectious Diseases and Immunology Department, Newcastle upon Tyne Hospitals Foundation Trust, Great North Children's Hospital, Newcastle upon Tyne, United Kingdom
[3] Paediatric Intensive Care Unit, Newcastle upon Tyne Hospitals Foundation Trust, Great North Children's Hospital, Newcastle upon Tyne, United Kingdom

NKUA Partner (Greece)

Principal investigator: Professor **Maria Tsolia**
Investigator/Research fellow: **Irini Eleftheriou**
Additional investigators (in alphabetical order):
Antonis Marmarinos, Kelly Syggelou, Maria Tampouratzi, Marietta Xagorari
Children's Hospital «P. And A. Kyriakou»,
2nd Department of Pediatrics,
Thivon and Levadias

Goudi, Athens

Micropathology Ltd :
Dr Marie Voice Post doc scientist
Professor Colin Fink , Clinical Microbiologist

EUCLIDS Austrian partner

PI: Werner Zenz[1]
Co-Investigators (in alphabetical order): Alexander Binder[1], Daniela S. Klobassa[1] Manfred Sagmeister[1], Nina A. Schweintzger[1]
[1]Department of General Paediatrics and Adolescent Medicine, Medical University of Graz, Graz, Austria
Austrian network, participating centers in Austria, Germany, Italy, Serbia, Lithuania (in alphabetical order):
Hinrich Baumgart[1], Markus Baumgartner[2], Uta Behrends[3], Ariane Biebl[4], Robert Birnbacher[5], Jan-Gerd Blanke[6], Carsten Boelke[7], Kai Breuling[3], Jürgen Brunner[8], Maria Buller[9], Peter Dahlem[10], Beate Dietrich[11], Ernst Eber[12], Johannes Elias[13], Josef Emhofer[2], Rosa Etschmaier[14], Sebastian Farr[15], Ylenia Girtler[16], Irina Grigorow[17], Konrad Heimann[18], Ulrike Ihm[19], Zdenek Jaros[20], Hermann Kalhoff[21], Wilhelm Kaulfersch[22], Christoph Kemen[23], Nina Klocker[24], Bernhard Köster[25], Benno Kohlmaier[26], Eleni Komini[27], Lydia Kramer[3], Antje Neubert[28], Daniel Ortner[29], Lydia Pescollderungg[16], Klaus Pfurtscheller[30], Karl Reiter[31], Goran Ristic[32], Siegfried Rödl[30], Andrea Sellner[26], Astrid Sonnleitne[r26], Matthias Sperl[33], Wolfgang Stelzl[34], Holger Till[1], Andreas Trobisch[26], Anne Vierzig[35], Ulrich Vogel[12], Christina Weingarten[36], Stefanie Welke[37], Andreas Wimmer[38], Uwe Wintergerst[39], Daniel Wüller[40], Andrew Zaunschirm[41], Ieva Ziuraite[42], Veslava Žukovskaja[42]
[1]Department of Pediatric and Adolescence Surgery, Division of General Pediatric Surgery, Medical University Graz, Austria
[2]Department of Pediatrics, General Hospital of Steyr, Austria
[3]Department of Pediatrics/Department of Pediatric Surgery, Technische Universität München (TUM), Munich, Germany
[4]Department of Pediatrics, Kepler University Clinic, Medical Faculty of the Johannes Kepler University, Linz, Austria
[5]Department of Pediatrics and Adolesecent Medicine LKH Villach, Austria
[6]Department of Pediatrics and Adolescent Medicine and Neonatology, Hospital Ludmillenstift, Meppen, Germany
[7]Hospital for Children's and Youth Medicine, Oberschwabenklinik, Ravensburg, Germany
[8]Department of Pediatrics, Medical University Innsbruck, Austria
[9]Clinic for Paediatrics and Adolescents Medicine, Sana Hanse-Klinikum Wismar, Germany
[10]Departement of Pediatrics, Medical Center Coburg, Germany
[11]University Medicine Rostock, Department of Pediatrics (UKJ), Rostock, Germany
[12]Department of Pulmonology, Medical University Graz, Austria
[13]Institute for Hygiene and Microbiology, University of Würzburg, Germany
[14]Clinical Institute of Medical and Chemical Laboratory Diagnostics, Medical University Graz, Austria
[15]Department of Pediatric Orthopedics and Adult Foot and Ankle Surgery, Orthopedic Hospital Speising, Vienna, Austria
[16]Department of Paediatrics, Regional Hospital Bolzano, Italy
[17]Department of Pediatrics and Adolescent Medicine, General Hospital Hochsteiermark/Leoben, Austria

[18]Department of Neonatology and Paediatric Intensive Care, Children's University Hospital, RWTH Aachen, Germany
[19]Paediatric Intensive Care Unit, Department of Paediatric Surgery, Donauspital Vienna, Austria
[20]Department of Pediatrics, General Public Hospital, Zwettl, Austria
[21]Pediatric Clinic Dortmund, Germany
[22]Department of Pediatrics and Adolescent Medicine, Klinikum Klagenfurt am Wörthersee, Klagenfurt, Austria
[23]Catholic Children's Hospital Wilhelmstift, Department of Pediatrics, Hamburg, Germany
[24]Department of Pediatrics, Krankenhaus Dornbirn, Austria
[25]Children's Hospital Luedenscheid, Maerkische Kliniken, Luedenscheid, Germany
[26]Department of General Paediatrics And Adolescent Medicine, Medical University of Graz, Graz, Austria
[27]Department of Paediatrics, Schwarzwald-Baar-Hospital, Villingen-Schwenningen, Germany
[28]Department of Paediatrics and Adolescents Medicine, University Hospital Erlangen, Germany
[29]Department of Pediatrics and Adolescent Medicine, Medical University of Salzburg, Austria
[30]Paediatric Intensive Care Unit, Medical University Graz, Austria
[31]Dr. von Hauner Children's Hospital, Ludwig-Maximilians- Universitaet, Munich, Germany
[32]Mother and Child Health Care Institute of Serbia, Belgrade, Serbia
[33]Department of Pediatric and Adolescence Surgery, Division of Pediatric Orthopedics, Medical University Graz, Austria
[34]Department of Pediatrics, Academic Teaching Hospital, Landeskrankenhaus Feldkirch, Austria
[35]University Children's Hospital, University of Cologne, Germany
[36]Department of Pediatrics and Adolescent Medicine Wilheminenspital, Vienna, Austria
[37]Department of Pediatric Surgery, Municipal Hospital Karlsruhe, Germany
[38]Hospital of the Sisters of Mercy Ried, Department of Pediatrics and Adolescent Medicine, Ried, Austria
[39]Hospital St. Josef, Braunau, Austria
[40]Christophorus Kliniken Coesfeld Clinic for Pediatrics, Coesfeld, Germany
[41]Department of Paediatrics, University Hospital Krems, Karl Landsteiner University of Health Sciences, Krems, Austria
[42]Children‘s Hospital, Affiliate of Vilnius University Hospital Santariskiu Klinikos, Lithuania

PERFORM Austrian Partner
PI: Werner Zenz[1]
Co-Investigators (in alphabetical order):
Daniela S. Kohlfuerst[1], Benno Kohlmaier[1], Manfred G. Sagmeister[1], Nina A. Schweintzger[1]
Clinical recruitment partners (in alphabetical order):
Sebastian Bauchinger[1], Hinrich Baumgart[4], Martin Benesch[3], Katharina Beitzke[1], Astrid Ceolotto[1], Ernst Eber[2], Siegfried Gallistl[1], Harald Haidl[1], Almuthe Hauer[1], Larissa Krenn[4], Klaus Pfurtscheller[4], Mirjam Pocivalnik[4], Tobias Niedrist[7], Siegfried Rödl[4], Andrea Skrabl-Baumgartner[1], Matthias Sperl[6], Holger Till[5], Andreas Trobisch[1], Rafael Ulreich[4], Maria Vittinghoff[8], Gerald Wendelin[1]Author Affiliations:
[1]Department of General Paediatrics and Adolescent Medicine, Medical University of Graz, Graz, Austria
[2]Department of Pediatric Pulmonology, Medical University of Graz, Graz, Austria
[3]Department of Pediatric Hematooncoloy, Medical University of Graz, Graz, Austria,
[4]Paediatric Intensive Care Unit Medical University of Graz, Graz, Austria

[5]Department of Paediatric and Adolescence Surgery, Medical University of Graz, Graz, Austria
[6]Department of Pediatric Orthopedics, Medical University of Graz, Graz, Austria
[7]Clinical Institute of Medical and Chemical Laboratory Diagnostics, Medical University of Graz, Graz, Austria
[8]Division of General Anaesthesiology, Emergency- and Intensive Care Medicine, Medical University of Graz, Graz, Austria

London School of Hygiene and Tropical Medicine
WP 5/WP1
Principal Investigator:
Dr Shunmay Yeung[1,2 ,3] PhD, MBBS, FRCPCH, MRCP, DTM&H
Research Group:
Dr Juan Emmanuel Dewez[1] MD, DTM&H, MSc
Mr David Bath[4] MSc, MAppFin, BA(Hons)
Dr Alec Miners[4] BA(Hons), MSc, PhD
Dr Ruud Nijman[3] PhD MSc MD MRCPCH
Department of Clinical Research, Faculty of Infectious and Tropical Disease, London School of Hygiene and Tropical Medicine, London, UK
Department of Global Health and Development, Faculty of Public Health and Policy, London School of Hygiene and Tropical Medicine, London, UK
St. Mary's Hospital Imperial College Hospital, London, UK
Department of Health Services Research and Policy, Faculty of Public Health and Policy, London School of Hygiene and Tropical Medicine, London, UK

Radboud University Medical Center (RUMC), The Netherlands

Principal Investigators:
Ronald de Groot [1], Michiel van der Flier [1,2], Marien I. de Jonge[1]

Co-investigators Radboud University Medical Center (in alphabetical order) :
Koen van Aerde[1,2], Wynand Alkema[1], Bryan van den Broek[1], Jolein Gloerich[1], Alain J. van Gool[1], Stefanie Henriet[1,2], Martijn Huijnen[1], Ria Philipsen[1], Esther Willems[1]

Investigators PeDBIG PERFORM DUTCH CLINICAL NETWORK (in alphabetical order):
G.P.J.M. Gerrits[7], J. Heidema [3], C.J. Miedema [4], C. Neeleman [1] C.C. Obihara [5], G.A. Tramper-Stranders [6]

1. Radboud University Medical Center, Nijmegen, The Netherlands
2. Amalia Children's Hospital, Nijmegen, The Netherlands
3. St. Antonius Hospital, Nieuwegein, The Netherlands
4. Catharina Hospital, Eindhoven, The Netherlands
5. ETZ Elisabeth, Tilburg, The Netherlands
6. Franciscus Gasthuis, Rotterdam, The Netherlands
7. Canisius Wilhelmina Hospital, Nijmegen, The Netherlands
We would also like to thank the GENDRES consortium:
GENDRES network (www.gendres.org)
This study received support from the Instituto de Salud Carlos III (Proyecto de Investigación en Salud, Acción Estratégica en Salud): project GePEM ISCIII/PI16/01478/Cofinanciado FEDER) and project ReSVinext ISCIII/PI16/01569/Cofinanciado FEDER (F.M.-T.);

Consellería de Sanidade, Xunta de Galicia (RHI07/2-intensificación actividad investigadora, PS09749 and 10PXIB918184PR), Instituto de Salud Carlos III (Intensificación de la actividad investigadora 2007–2012, PI16/01569), Fondo de Investigación Sanitaria (FIS; PI070069/PI1000540) del Plan Nacional de I + D + I and 'Fondos FEDER' (F.M.-T.), and 2016-PG071 Consolidación e Estructuración REDES 2016GI-1344 G3VIP (Grupo Gallego de Genética Vacunas Infecciones y Pediatría, ED341D R2016/021) (F.M.T). We would like to gratefully acknowledge the collaboration of the Translational Research Network in Pediatric Infectious Diseases (RITIP—www.ritip.org) and their members with GENDRES. GENDRES research group (Genetics, Vitamin D and Respiratory Infections Research Network): www.gendres.org. Further details may be found at www.gendres.org. The following are members of GENDRES Network: Unidade de Xenética, Departamento de Anatomía Patolóxica e Ciencias Forenses, Instituto de Ciencias Forenses, Facultade de Medicina, Universidade de Santiago de Compostela, 15782 Santiago de Compostela, Spain: Ruth Barral-Arca, Sara Pischedda; GenPoB Research Group, Instituto de Investigaciones Sanitarias (IDIS), 15706 Santiago de Compostela, Spain: Antonio Justicia Grande, Beatriz Morillo, Lorenzo Redondo Collazo, Carmen Rodríguez-Tenreiro, Ruth Barral-Arca, Sara Pischedda, Nazareth Martinón-Torres, José María Martinón Sánchez;Área Asistencial Integrada de Pediatría, Hospital Clínico Universitario, Santiago de Compostela, Galicia, Spain: José Peña Guitián, Carmen Curros Novo, Miriam Puente Puig, Rosaura Leis Trabazo, Nazareth Martinón-Torres, José María Martinón Sánchez; Biobank, Servicio Anatomía Patológica, Hospital Clínico Universitario, Santiago de Compostela, Galicia, Spain: Máximo Francisco Fraga Rodríguez, José Ramón Antúnez; Servicio de Pediatría, Hospital Materno Infantil Virgen del Camino, Pamplona, Navarra, Spain: Enrique Bernaola Iturbe, Laura Moreno Galarraga, Jorge Álvarez; Departamento de Pediatría, Complejo Hospitalario Universitario de Orense, Galicia, Spain: Teresa González López, Delfina Suarez Vázquez, Ángela Vázquez Vázquez, Susana Rey García; Servicio de Pediatría, Hospital de la Inmaculada de Granada, Andalucía, Spain: Francisco Giménez Sánchez; Servicio de Pediatría, Hospital Torrecárdenas, Almería, Andalucía, Spain: Miguel Sánchez Forte; Servicio de Pediatría, Hospital La Paz, Madrid, Spain: Cristina Calvo Rey; Servicio de Pediatría, Hospital Severo Ochoa de Madrid, Spain: María Luz García García; Servicio de Pediatría, Complejo Asistencial Universitario de León, Castilla-León, Spain: Ignacio Oulego Erroz, David Naranjo Vivas, Santiago Lapeña, Paula Alonso Quintela, Jorge Martínez Sáenz de Jubera, Estibaliz Garrido García; Servicio de Pediatría, Hospital de Donostia, San Sebastián, País Vasco, Spain: Cristina Calvo Monge, Eider Oñate Vergara; Servicio de Pediatría, Complejo Hospitalario de Jaén, Andalucía, Spain: Jesús de la Cruz Moreno, Mª Carmen Martínez Padilla; Servicio de Pediatría, Hospital Quirón, Málaga,
Andalucía, Spain: Manuel Baca Cots; Servicio de Pediatría, Hospital Carlos Haya, Málaga, Andalucía, Spain: David Moreno Pérez; Servicio de Pediatría, Hospital Virgen de la Arrixaca, Murcia, Spain: Susana Beatriz Reyes, María Cruz León León.

Genetic Determinants of Kawasaki Disease Study group (UK) ;

Chief Investigator: Professor M Levin
Study co-ordinator: R Galassini
Addenbrookes Hospital, Cambridge: Dr Y Singh (PI), J Bytham
Airedale General Hospital PI Dr P Bala: A Kitching
Alder Hey children's Hospital: Dr S Paulus (PI), Prof E Carol (PI), Dr B Larru (PI). S Wadeson, J Johnstone, R Jennings
Birmingham Children's Hospital; Dr A Chickermane (PI), Cotter
Bradford Royal Infirmary: Dr H Jepps (PI); T Booth, R Swingler
Bristol Royal Infirmary: Prof R Tulloh (PI; Karen Sheehan
Burton Hospital: Dr M Ahmed (PI), S Boswell, C Backhouse

Calderdale Royal Hospital Dr M Olabi (PI), KU Rahman (PI), S Kilroy, M Home
Durham & Darlington NHS Trust: Dr T Banerjee (PI), Dr G Nyamugunduru (PI), A Cowton, D Egginton
East Surrey Hospital; Dr M Jawad (PI)L Bailey
Evelina Children's Hospital: Dr E Menson (PI)
Great Ormond Street Hospital, Dr P Brogan (PI), Y Glackin
Harrogate Hospital: Miss C Brunskill (PI)
Heartlands Hospital Dr S Hackett (PI), J Daglish
Hereford County Hospital Dr S Meyrick (PI), E Collins
Hull University Teaching Hospital, Mr D Bolton (PI)
Imperial College Healthcare NHS Trust; Dr J Herberg (PI), S Gormley, S Mustafa
Ipswich Hospital: Dr P Desai (PI) L Hunt
Kingston Hospital Dr T Chawatama (PI), Dr S Luck (PI), J Crooks, T O'Brien
Leeds General Infimary Dr S O'Riordan (PI), **N Balatoni, N Maher**
Macclesfield General Hospital: Dr Chandrasekaran (PI), N Keenan
New Cross Hospital, Wolverhampton, Dr K Davies (PI), S Kempson, C Busby
North Manchester General Hospital: Dr E Odeka (PI), G O'Connor
North Tees & Hartlepool Dr I Haar (PI), G Osborne, H Walker
Northwick Park Hopsital: Dr A Williams (PI)
Oldham Hospital Dr E Odeka (PI), L Woodward, C Rishton
Peterborough City Hospital; Dr V Puthi (PI), A Pearson, P Goodyear
Pinderfields General Hospital, Dr C Davidson (PI), Dr N De Vere (PI), G Castle
Royal Albert Edward Infirmary, Dr M Farrier (PI), N Pemberton
Royal Bolton Hospital: PI Dr S Misra, C Fish, P Graham, J Henry
Royal Lancaster Infirmary, Dr A Olabi (PI) K Allison
Royal Shrewsbury Hospital: Dr A Kannivelu (PI), Mr J Jones (PI)
Royal Stoke University Hospital: Dr J Alexander (PI) E Roe, R Pringle, A Cope
Sheffield Children's Hospital, Dr F Shackley (PI), S Gormley
South Tees Hospital: Dr R Kumar (PI), GMcilhinney, S Armstrong
St George's Hospital, Tooting, Prof P Heath (PI), E Vitale, J Stuart
St Richard's Hospital, Chichester: Dr N Brennan (PI), S Floyd
Stepping Hill Hospital, Stockport, Dr C Cooper, S Bennett
Tameside Hospital, Dr A Petkar (PI), Dr C Greenway (PI), W Hulse
The Royal Alexandra Hospital, Brighton; Dr K Fidler (PI), K Moscovici, S Sobowieck Kouman
The Royal Brompton Hospital, Dr F Franklin (PI), Dr M Bartsota
The Royal Cornwall Hospital, Dr N Venkata (PI), Dr A Prendiville (PI), Dr O Elmasry (PI), Mrs H Osborne (PI), G Craig, B Bromage
Torbay Hospital, Dr M Raman (PI), Ms P Fitzell (PI), H Bearne, J Palmer
UK Kawasaki Support Group Mrs Sue Davidson
University Hospital, Coventry & Warwickshire, Dr M Rajimwale (PI), S Dale
West Middlesex Hospital: Dr J Rangasami (PI), Ar Sayan
Worthing Hospital: Dr N Brennan (PI), G Hobden
York Hospital: Dr M Wheeler, A Clayton, B Coop
