# Supplementary figures and images for: Biomarkers for the Discrimination of Acute Kawasaki Disease From Infections in Childhood
Source: Front Pediatr. 2020 Jul 22;8:355. doi: 10.3389/fped.2020.00355 (PMC7388698; doi:10.3389/fped.2020.00355)

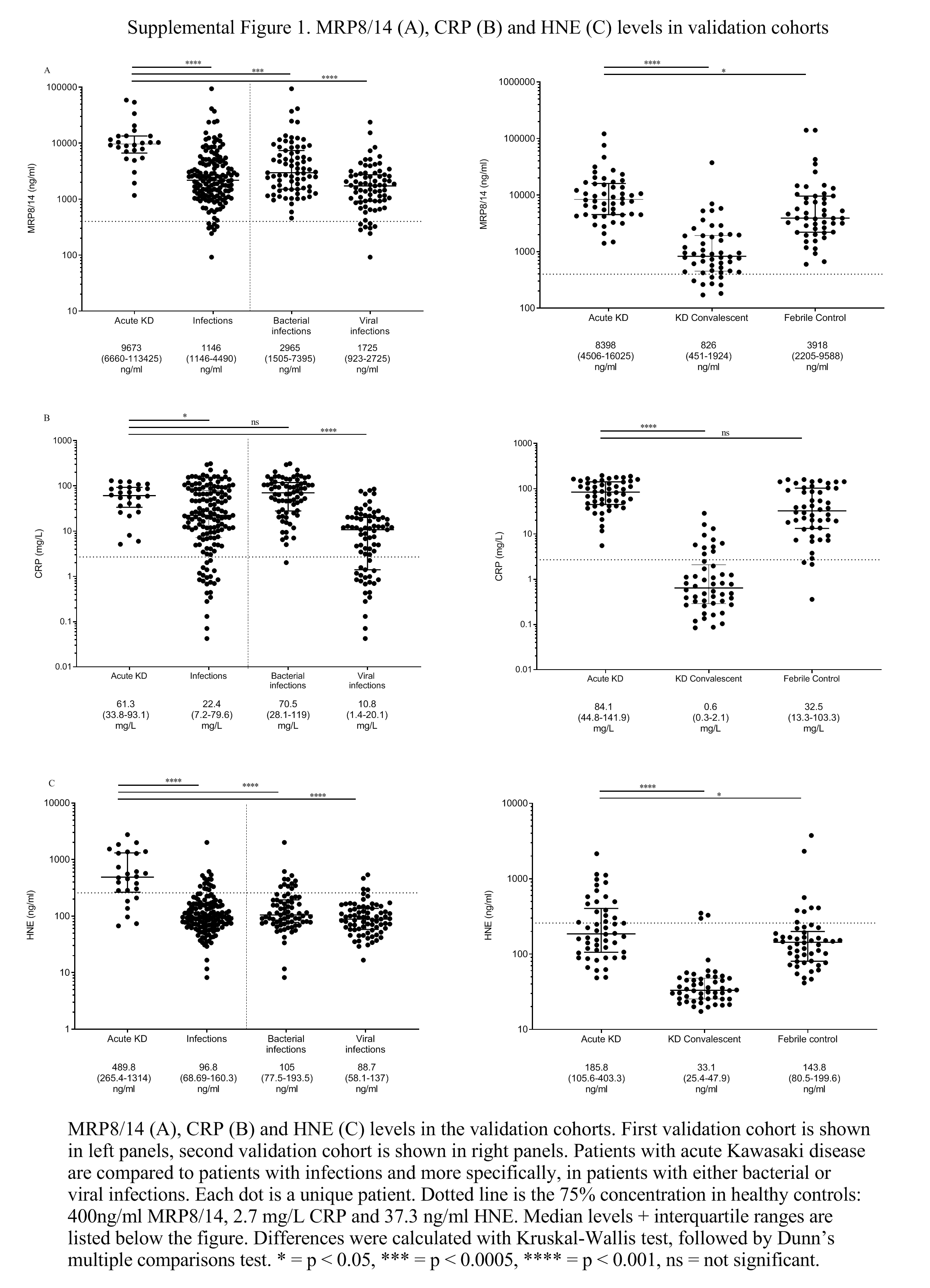

Supplement: Supplementary file 3 [file Image_1.TIF]

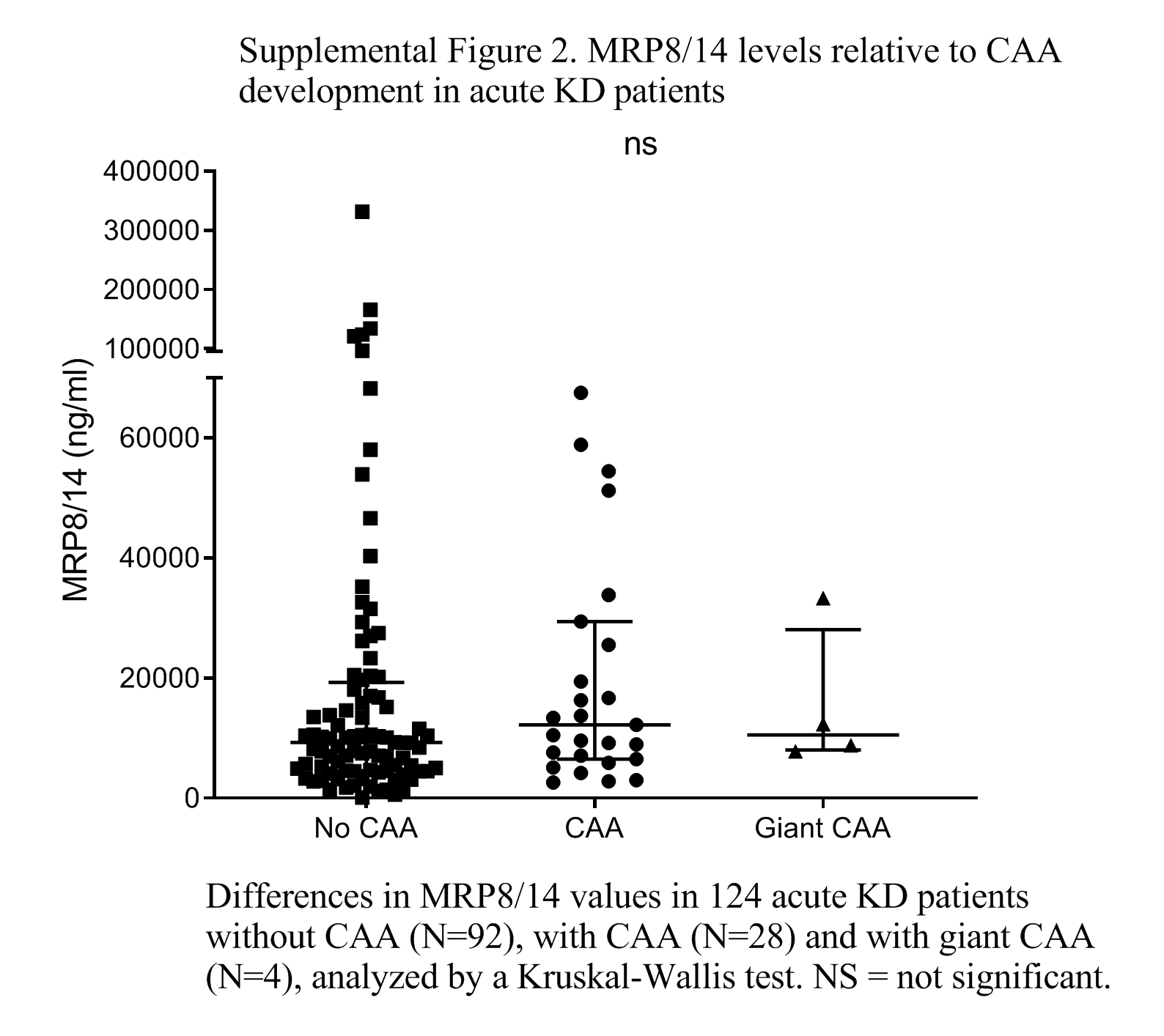

Supplement: Supplementary file 4 [file Image_2.TIF]
